# Ecological factors related to the widespread distribution of sylvatic *Rhodnius ecuadoriensis *populations in southern Ecuador

**DOI:** 10.1186/1756-3305-5-17

**Published:** 2012-01-13

**Authors:** Mario J Grijalva, Victoria Suarez-Davalos, Anita G Villacis, Sofia Ocaña-Mayorga, Olivier Dangles

**Affiliations:** 1 Tropical Disease Institute, Department of Biomedical Sciences, Heritage College of Osteopathic Medicine, Ohio University, Athens OH USA; 2 Center for Infectious Disease Research, Pontifical Catholic University of Ecuador, Quito, Ecuador; 3 IRD UR072, LEGS UPR 9034, CNRS 91198 Gif-sur Yvette Cedex and Université Paris-Sud 11, France

**Keywords:** *Rhodnius ecuadoriensis*, sylvatic triatomines, Ecuador, Chagas disease

## Abstract

**Background:**

Chagas disease transmission risk is a function of the presence of triatomines in domestic habitats. *Rhodnius ecuadoriensis *is one of the main vectors implicated in transmission of *Trypanosoma cruzi *in Ecuador. This triatomine species is present in domestic, peridomestic and sylvatic habitats in the country. To determine the distribution of sylvatic populations of *R. ecuadoriensis *and the factors related to this distribution, triatomine searches were conducted between 2005 and 2009 in southern Ecuador.

**Methods:**

Manual triatomine searches were conducted by skilled bug collectors in 23 communities. Sylvatic searched sites were selected by a) directed sampling, where microhabitats were selected by the searchers and b) random sampling, where sampling points where randomly generated. Domiciliary triatomine searches were conducted using the one man-hour method. Natural trypanosome infection was determined by microscopic examination and PCR. Generalized linear models were used to test the effect of environmental factors on the presence of sylvatic triatomines.

**Results:**

In total, 1,923 sylvatic individuals were collected representing a sampling effort of 751 man-hours. Collected sylvatic triatomines were associated with mammal and bird nests. The 1,219 sampled nests presented an infestation index of 11.9%, a crowding of 13 bugs per infested nest, and a colonization of 80% of the nests. Triatomine abundance was significantly higher in squirrel (*Sciurus stramineus*) nests located above five meters from ground level and close to the houses. In addition, 8.5% of the 820 examined houses in the same localities were infested with triatomines. There was a significant correlation between *R. ecuadoriensis *infestation rates found in sylvatic and synanthropic environments within communities (*p *= 0.012). Parasitological analysis revealed that 64.7% and 15.7% of the sylvatic bugs examined (n = 300) were infected with *Trypanosoma cruzi *and *T. rangeli *respectively, and 8% of the bugs presented mixed infections.

**Conclusions:**

The wide distribution of sylvatic *R. ecuadoriensis *populations may jeopardize the effectiveness of control campaigns conducted to eliminate domestic populations of this species. Also, the high *T. cruzi *infection rates found in sylvatic *R. ecuadoriensis *populations in southern Ecuador could constitute a risk for house re-infestation and persistent long-term Chagas disease transmission in the region.

## Background

Chagas disease is caused by the hemoflagellate protozoan parasite *Trypanosoma cruzi *and is transmitted mainly by the feces of infected triatomine bugs and by blood transfusions [1]. At present, there are 140 extant species formally recognized in the Triatominae subfamily [2]. While all of these are probably capable of transmitting *T. cruzi*, relatively few species show epidemiological importance as vectors of *T. cruzi *to humans [2]. However, most species maintain enzootic cycles involving wild mammals in a variety of biotopes [3,4]. Control of the disease has mostly relied on eliminating domestic vector populations [3]; however, the re-infestation of insecticide-treated households by wild vectors is common [5]. Furthermore, sylvatic populations actively transmit the disease in wide regions of the Americas [6,7]. The control of re-infestation of households by wild vectors may therefore be unattainable unless sustained and frequent practices combining several vector control approaches are applied. Traditional approaches that involve spraying households with residual insecticides are unlikely to be effective against triatomines living in sylvatic habitats [3,8,9]. Increasing reports of sylvatic triatomine species invading and sometimes colonizing peridomestic and domestic habitats justifies research on their original wild populations and habitats [5,9,10].

In Ecuador, sixteen triatomine species have been reported [11], and it is believed that at least 13 are vectors or potential vectors of Chagas disease [11,12]. *Rhodnius ecuadoriensis *is an important vector in Ecuador [9,13,14]. Studies related to its ecology, feeding, and defecation patterns indicate that this species has bionomic traits of an efficient *T. cruzi *vector [14]. *R. ecuadoriensis *is bivoltine and colonizes domestic, peridomestic, and sylvatic habitats. The domestic populations of *R. ecuadoriensis*, considered to be native to western Ecuador, extend from coastal and southern Ecuador to northern Perú [11,13,15]. Along the central coastal region of Ecuador and in the subtropical valleys of Santo Domingo de los Tsáchilas Province, its sylvatic habitat has been associated with the endemic palm *Phytelephas aequatorialis *[11] and other plant species [16]. This species has been found in association with various vertebrate hosts, including squirrels [*Sciurus stramineus *(Rodentia: Sciuridae)], birds [*Campylorhynchus fasciatus *(Passeriformes: Troglodytidae)], opossums [*Didelphis marsupialis *(Didelphimorphia; Didelphidae], and mice [*Mus musculus *(Rodentia: Muridae)] [9,16-18]. In southern Ecuador, *R. ecuadoriensis *was believed to be located exclusively in domestic areas [11,13], where palm trees are absent, making vector control by means of house spraying feasible. However, the recent report of sylvatic *R. ecuadoriensis *associated with squirrel nests (*S. stramineus*) in southern Ecuador [18] illustrates the need of a new approach for controlling *R. ecuadoriensis *in this region.

The present study aimed to 1) describe the distribution of sylvatic *R. ecuadoriensis *populations in southern Ecuador, 2) identify some ecological factors associated with their abundance in sylvatic habitats, 3) determine their rate of infection with trypanosomes, and 4) discuss the implication of these findings for long-term Chagas disease control efforts in the region.

## Methods

### Study area

**Figure 1:**
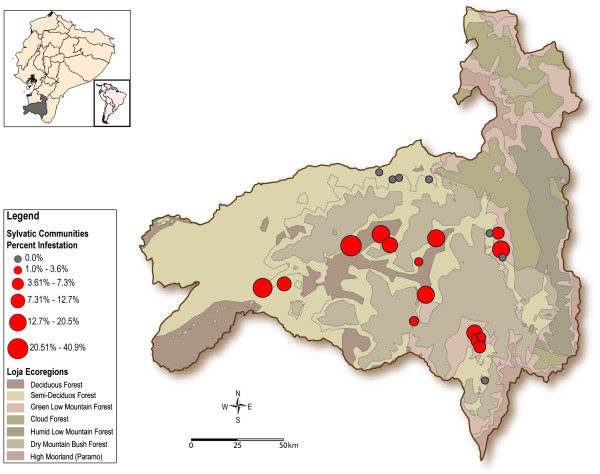
**Map of Loja Province showing the vegetation zones and the location of the studied communities**. Red circles indicate localities that presented sylvatic triatomine infestation. Diameter of the circles indicates the percentage of sylvatic microhabitat infestation. Vegetation zones as Sierra et al. (1999).

From 2005 to 2009 searches of sylvatic triatomines were conducted in 23 communities located in eight counties in Loja Province (Figure 1, Table 1). The sampled microhabitats ranged from 640 to 1,958 m.a.s.l. and included four vegetation zones (deciduous forest, semi-deciduous forest, green low mountain forest and dry mountain bush forest) [19]. This province has a dry, temperate climate, and the vegetation is dominated by bushes and prickly and herbaceous plants [19]. The main agricultural crops of the region are corn, kidney beans, yucca, papaya, peanuts, bananas, and coffee. The temperature and relative humidity in the sylvatic sites sampled were determined by using LogTag Recorders (Trix-8, Hong Kong, China). Five of these recorders were set for one day to record temperature and relative humidity every hour in selected microhabitats.

**Table 1:** Entomological indices of *R. ecuadoriensis *collected in domestic/peridomestic and sylvatic habitats from 23 rural communities located in Loja province, Ecuador

		**Domicile/peridomicile**				**Sylvatic**			
County	Community	Infestation index % (n)	Density	Crowding	Colonization index (%)	Infestation index % (n)	Density	Crowding	Colonization index (%)
Calvas	Chaquizhca	32.3 (31)	1.2	3.7	40	20.5 (73)	1.3	6.5	60
	Jacapo	0 (53)	-	-	-	1.8 (56)	0.02	1	100

Catamayo	La Extensa^a^	4.3 (47)	8.2	193.5	100	7.3 (41)	0.1	1	100
	Santa Rita	0 (20)	-	-	-	0 (22)	-	-	-

Celica	El Guineo^a^	0 (17)	-	-	-	12.5 (48)	1.1	87	83
	La Ciénega^a^	21.4 (42)	12.4	58	78	17.4 (144)	2.7	15.6	72

Chaguarpamba	Amarillos^a^	0 (39)	-	-	-	0 (37)	-	-	-
	Jorupe	0 (26)	-	-	-	0 (24)	-	-	-
	Lozumbe	0 (25)	-	-	-	0 (35)	-	-	-
	Mizquillana	0 (29)	-	-	-	0 (10)	-	-	-
	Venecia	0 (21)	-	-	-	0 (43)	-	-	-

Espíndola	Tundurama	1.6 (61)	0.02	1	0	0 (8)	-	-	-

Gonzanamá	Algarrobera	0 (27)	-	-	-	0 (9)	-	-	-
	San Jacinto^a^	9.1 (11)	6.4	70	100	17.4 (190)	3.5	20.4	82

Paltas	Ashimingo	17.2 (29)	8.4	48.6	80	20 (5)	2.2	11	100
	Bramaderos^a^	19.7 (61)	12.4	62.9	100	40.9 (66)	4.1	10.1	85
	Coamine^a^	10.9 (46)	28	257.4	80	1.5 (67)	0.1	6	100
	Las Cochas	0 (76)	-	-	-	17.4 (23)	0.9	5.3	75
	Naranjo Dulce^a^	10.8 (37)	0.6	5.8	75	12.7 (71)	1	8.1	89

Quilanga	Galápagos^a^	29.4 (34)	21.6	73.3	100	12 (117)	1.3	10.71	86
	Jacapo Q^a^	0 (22)	-	-	-	3.5 (85)	1.7	47.3	75
	Santa Rosa	15.4 (39)	5.2	33.7	100	5.6 (36)	0.6	11	100
	Tuburo	18.5 (27)	33.5	181	100	11.1 (9)	1	-	100

**Total**		**8.5 (820)**	**6.3**	**73.8**	**83**	**11.9 (1,219)**	**1.6**	**13.3**	**80**

^a ^communities where random sampling was conducted

### Triatomine sampling

Manual searches for triatomines were conducted in sylvatic habitats in the 23 communities (Table 1) by skilled bug collectors. These searches resulted in an effort of ~751 man-hours. Microhabitats within these sites--such as animal nests, burrows, tree holes and the ground beneath sticks--were selected and examined once by the searcher. Searched sites were selected by a) directed sampling, where microhabitats were selected by the searchers and b) random sampling, where sampling points where randomly generated.

#### Directed sampling

This sampling method was used in 13 communities and allowed searchers to sample areas where it was difficult to locate quadrats or transects, such as river banks and areas between mountains and ravines as previously described [18].

#### Random sampling

Manual searches were performed in randomly generated sampling points inside 600 m^2^-quadrats located in ten communities, as previously described [16]. The sites were selected for the high prevalence of triatomine-infested homes and the presence of vegetation patches in the vicinity of houses.

#### Domicile/Peridomicile searches

All households in each community were visited. Informed consent was obtained from the head of the household according to a protocol approved by Ohio University and Catholic University of Ecuador Institutional Review Boards. Manual triatomine searches were conducted using the one man-hour method as previously described [13].

Triatomines found in each microhabitat were collected in individually labeled plastic containers and taken to the Insectary at the Center for Infectious Diseases Research, Catholic University of Ecuador, Quito, Ecuador. The specimens were then counted, classified by instar, and identified to species based on morphological criteria [12,20] and by comparisons to specimens stored at the Entomology Museum of the Catholic University (QCAZ) in Quito, Ecuador.

### Entomological indexes

The indexes were calculated for each community sampled and for different microhabitat types found in domiciles and sylvatic areas. The indexes calculated included infestation index (100 × number of nests or domiciles infested/number of nests or domiciles examined), density (number of triatomines collected/number of nests or domiciles examined), crowding (number of triatomines collected/number of nests or domiciles infested) and colonization index (100 × number of nests or domiciles with nymphs/number of nests or domiciles infested) [1,16].

### Generalized linear models (GLMs)

We used GLMs with a log link to test the effect of environmental factors (type of nest, height from ground level, and distance to the nearest house) and the different combinations of these factors on the number of triatomines found in each random sampling point of the 10 sites where 600 m^2 ^quadrats were sampled. The non-random samples were not included in this analysis due to the bias introduced by the searcher during the directed sampling. We used the value of Akaike's Information Criterion (AIC) [21] to study the effect of removing each of the selected ecological factors from the model. Likelihood ratio tests (LRT) were used to test whether the suppression of each factor significantly affects the fit of the model. These analyses were performed using the mass library for R [22].

### Correlation between sylvatic and domestic triatomine infestation

Spearman's rank correlation coefficient (rho) was used to study the correlation between triatomine infestation in domiciles and triatomine infestation in sylvatic habitats observed in a locality.

### Natural Trypanosome infection

A total of 300 sylvatic triatomines (nymphs III, IV, V and adults) was selected randomly for parasitological analysis. Triatomines were washed in White's solution (HgCl 0.8 mM, NaCl 111 mM, HCl 0.125%, and 25% v/v of ethanol 95%) before being dissected under a stereomicroscope. Feces and intestinal content were mixed with 200 μl of sterile PBS. One aliquot was used for microscopic examination to detect flagellates and a second aliquot was stored at -20°C for DNA extraction. DNA was extracted with a DNeasy kit (Qiagen, Valencia, CA) following manufacturer's protocol. The presence of trypanosomatid DNA was determined by PCR amplification using the S35/S36 primer set [23]. Stock strain *T. cruzi *and *T. rangeli *DNA were used as positive controls and distilled water was used as negative control in each PCR run. The infection index (100 × number of infected individuals/total number of analyzed individuals) was calculated for each type of microhabitat.

## Results

### Sylvatic triatomines

**Figure 2:**
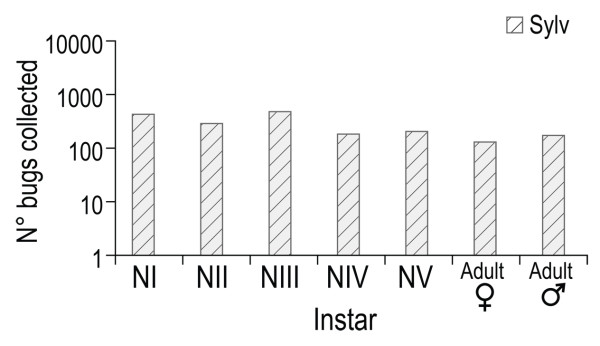
**Population structure of triatomines collected in sylvatic habitats of 15 localities of Loja province**.

In total, 1,923 *R. ecuadoriensis *specimens were collected in sylvatic habitats from 15 out of 23 localities sampled in Loja Province (Figure 1). The specimens collected belonged to all nymphal stages and adults, with more individuals at the NI and NIII stages (Figure 2). Sylvatic *R. ecuadoriensis *populations were found at altitudes ranging from 661 to 1,663 meters above sea level (m.a.s.l.) and comprising the four sampled vegetation zones (deciduous forest, semi-deciduous forest, green low mountain forest, and dry mountain bush forest). Microhabitat temperature ranged from 15.1°C to 31.7°C (average of 21.4°C) and the relative humidity from 32.8% to 100% (average of 86.2%).

### Domestic triatomines

In domiciliary habitats, a total of 5,165 *R. ecuadoriensis *was collected in 12 of the 23 sampled localities (Table 1). In addition, domiciles were found infested with *Triatoma carrioni, Panstrongylus chinai*, and *P. rufotuberculatus *(data not shown). However, we found no sylvatic populations of these three species.

### Entomological Indexes

*R. ecuadoriensis *specimens were found in 11.9% of the 1,219 sylvatic nests and in 8.5% of the 820 domiciles that were examined. The overall triatomine density was 1.6 bugs per nest and 6.3 insects per domicile examined (Table 1). Triatomine crowding was 13.3 bugs per infested nest and 73.8 bugs per infested domicile. Finally, *R. ecuadoriensis *nymphs were collected in 80% of the infested nests and 83% of the domiciles.

### Microhabitats of sylvatic triatomines

Random searches within quadrats revealed that squirrel nests (*S. stramineus*) had the highest infestation index (21.1%, n = 71), followed by mouse/rat nests (9.4%, n = 74) and bird nests (*Campylorhynchus fasciatus *1.4%, n = 148) (Table 2). No triatomines were found in opossum (n = 2) and armadillo (n = 25) burrows, under rocks (n = 32), dead tree trunks (n = 118), or between leaves and roots of plants [*Musa paradisiaca *(n = 67), *Zea mays *(n = 7), and orchid (n = 2) and bromelia (n = 1) species]. Directed sampling showed triatomine infestation in 22.9% mouse/rat nests (n = 131), followed by a 19.9% triatomine infestation in squirrel nests (n = 276), 12.5% in opossum nests (n = 8) and 3.3% in bird nests (n = 390). No triatomines were found in armadillo (n = 7) or skunk (n = 2) burrows sampled.

**Table 2:** Infestation index by nest types found random searches in quadrats sampled in 10 rural communities located in Loja Province, Ecuador

	Type nest								
	
Community	Squirrel			Mouse/rat			Bird		
	
	No. examined	No. infested	Infestation index (%)	No. examined	No. infested	Infestation index (%)	No. examined	No. infested	Infestation index (%)
Amarillos	12	-	-	7	-	-	4	-	-
Bramaderos	11	6	54.5	4	2	50	3	-	-
Coamine	-	-	-	4	-	-	18	-	-
El Guineo	5	2	40	5	-	-	5	-	-
Galápagos	4	-	-	8	3	37.5	11	-	-
Jacapo Q	6	-	-	15	1	6.7	8	-	-
La Ciénega	17	4	23.5	2	-	-	22	-	-
La Extensa	-	-	-	4	-	-	13	1	7.7
Naranjo Dulce	16	3	18.8	16	1	6.2	19	1	5.3
San Jacinto	-	-	-	7	-	-	44	-	-

**Total**	71	15	21.1	74	7	9.4	148	2	1.4

### Factors influencing triatomine abundance

**Figure 3:**
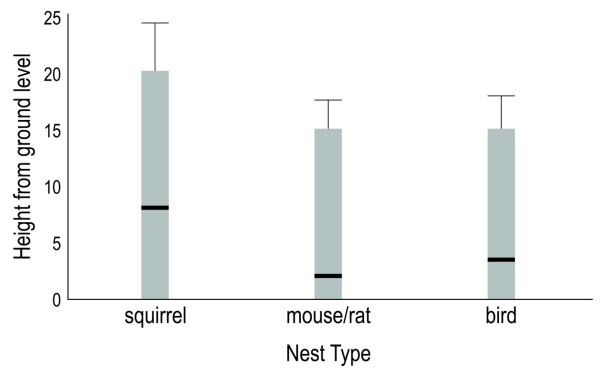
**Heights from ground level of tree types of nests sampled in 10 sylvatic quadrats located in Loja province**. Black line shows the mean height and error bars show the standard deviation.

Generalized linear model (GLM) analysis performed on data from the random searches revealed that the factors affecting the abundance of triatomines in sampled microhabitats included type of nest, nest's height from ground level, nest's distance to nearest house, and the combination type of nest × height from ground level (Table 3, Figure 3). While all these factors significantly explained observed variations in triatomine abundance (P < 0.001), removing the "type of nest" factor from the overall GLM model caused the highest increase in AIC. This suggests that nest type is one of the main drivers of triatomine abundance in sylvatic habitats.

**Table 3:** Results of the Generalized Linear Model's analysis on triatomine samplings

Effect	Terms included in the model	AIC	ΔAIC	P
all	all	1315		< 0.001
nest*height	all	1315.8	0.8	< 0.001
height	all	1320.1	5.1	< 0.001
distance	all	1375.4	60.4	< 0.001
nest	all	1388.3	73.3	< 0.001

Akaike's Information Criterion (AIC) is given to test for the significance of selected ecological factors on triatomine infestation. The factors or combination of factors that presented change in AIC value are listed

Additionally, there was a significant correlation between the triatomine infestation index found in domiciliary units and in sylvatic habitats within the same locality (Spearman's rho = 0.507, *p *= 0.012).

### Natural *Trypanosoma *infection in sylvatic *R. ecuadoriensis*

A total of 300 triatomines collected in different sylvatic habitats from 13 communities in 6 counties was analyzed parasitologically (Table 4). PCR showed that 72.3% of the sylvatic *R. ecuadoriensis *were infected with trypanosomatids. Of these, 56.7% and 7.7% presented single infection with *T. cruzi *and *T. rangeli*, respectively, and 8% presented mixed infections with *T. cruzi *and *T. rangeli *(Table 4). The parasite *T. cruzi *was found in 10 out of 13 communities and in at least one community in each county indicating widespread distribution of infected *R. ecuadoriensis *in sylvatic habitats. Triatomines collected in squirrel nest microhabitat presented the highest *T. cruzi *infection index (73.4%, n = 177 bugs), followed by rat/mice nests (53.6%, n = 112) (Table 4). Although triatomines collected in bird nests accounted only for 3.7% of the total analyzed bugs, infection with *T. cruzi *was found 36.4% of them (2 NIII and 2 adult males out of 13 analyzed individuals). Conversely, *T. rangeli *was found in bugs collected in rat/mouse nests (19.6%) and squirrel nests (14.1%). No *T. rangeli *infected bugs were collected in bird nests (Table 4). Natural infection with *T. cruzi *was found to be higher in adults than in nymphs and appears to increase with developmental stage. Conversely, *T. rangeli *infection was higher in nymphs than in adults (Table 5).

**Table 4:** Natural trypanosomatid infection^a ^in sylvatic *R. ecuadoriensis *collected in 13 localities from Loja province, Ecuador

County	Comunity	Microhabitat (n)^b^	% *T. cruzi*	% *T. rangeli*	Mixed infections
Calvas	Chaquizhca	Squirrel nest (15)	80.0	6.7	-
		Rat/mouse nest (6)	-	-	-
		Bird nest (2)	50.0	-	-

Catamayo	La Extensa	Bird nest (2)	50.0	-	-

Celica	La Ciénega	Squirrel nest (11)	72.7	-	-
		Rat/mouse nest (4)	-	-	-
	El Guineo	Squirrel nest (17)	35.3	11.8	-

Gonzanamá	San Jacinto	Squirrel nest (38)	78.9	-	7.9
		Rat/mouse nest (55)	63.6	9.1	12.7
		Bird nest (2)	50.0	-	-

Paltas	Ashimingo	Rat nest (1)	-	-	-
	Bramaderos	Squirrel nest (57)	61.4	8.8	15.8
		Rat/mouse nest (27)	40.7	29.6	7.4
	Coamine	Squirrel nest (6)	100.0	-	-
	Las Cochas	Squirrel nest (2)	50.0	-	-
	Naranjo Dulce	Squirrel nest (6)	16.7	-	-

Quilanga	Galápagos	Squirrel nest (17)	58.8	11.8	11.8
		Rat/mouse nest (19)	26.3	-	-
		Bird nest (5)	20.0	-	-
	Jacapo Q	Squirrel nest (7)	85.7	-	14.3
	Tuburo	Squirrel nest (1)	-	-	-
**All communities**		Rat/mouse nest	45.5	11.6	8.0
		Squirrel nest	65.0	5.6	8.5
		Bird nest	36.4	7.7	-

**TOTAL**		**All microhabitats (300)**	**56.7**	**7.7**	**8**

^a ^Determined by PCR analysis.

^b ^Number of triatomines analyzed from each type of microhabitat

**Table 5:** Natural *T. cruzi *and *T. rangeli *infection^a ^in relationship to the population structure of 300 sylvatic *R. ecuadoriensis *collected in 13 localities from Loja province, Ecuador

Triatomine Instar	n	*T. cruzi *only %	*T. rangeli *only %	*T. cruzi *&*T. rangeli *mixed %
**Male**	83	67.5	4.8	7.2
**Female**	76	69.7	0.0	9.2
**N V**	53	50.9	11.3	17.0
**N IV**	40	42.5	17.5	2.5
**N III**	48	35.4	12.5	2.1

**Total**	300	56.7	7.7	8.0

^a ^Determined by PCR analysis.

## Discussion

*R. ecuadoriensis *is widely distributed in southern and western Ecuador and north-western Peru and is considered an important *T. cruzi *vector. The presence of sylvatic triatomine populations of this species [18] may pose a problem to Chagas disease control efforts in domestic environments, especially when these populations have marked synanthropic tendencies and could potentially re-infest domestic habitats after spraying campaigns from sylvatic environments [13].

### Sylvatic population of *R. ecuadoriensis *in southern Ecuador

Our results showed 1) a wide distribution of sylvatic populations of *R. ecuadoriensis *throughout Loja Province and 2) a considerable infestation of rodent nests, which are highly abundant in the region. In the studied communities, the sylvatic distribution and abundance of this species seems to follow closely household infestation. Earlier reports indicated that *R. ecuadoriensis *was restricted to domestic and peridomestic habitats in Loja Province and northern Peru [11,24,25]. If so, spraying homes with insecticide could have been an effective control strategy. Moreover, previous reports from Peru suggested that *R. ecuadoriensis *had no sylvatic ecotypes, its presence being more related to passive transportation with humans [24,25]. In Ecuador, a pilot project including spraying efforts in 25 communities in the Loja province showed a reduction in triatomine infestation in domicile and peridomicile habitats (Grijalva et al. unpublished data). However, some communities with high household infestation presented continuous re-infestation after spraying. The high number of *R. ecuadoriensis *collected in sylvatic habitats located close to the dwellings could be the source of this re-infestation in domicile and peridomicile habitats. However, detailed studies of patterns and sources of re-infestation are still needed to determine the importance of sylvatic *R. ecuadoriensis *populations in Chagas disease transmission in Ecuador.

Additionally, the distribution of sylvatic *R. ecuadoriensis *closely depends on ecological associations. Sylvatic populations of species from the *pallescens *complex, which includes *R. pallescens*, *R. colombiensis*, and *R. ecuadoriensis*, have been found in wild habitats associated with palm trees. The first two species were particularly associated with *Attalea butyracea *[12,20,26,27] while *R. ecuadoriensis *has been associated with *Phytelephas *palm trees [11]. However, our results indicate that *R. ecuadoriensis *presence appears to be more associated to vertebrate hosts distribution than to any given species of plant [16-18].

### Factors influencing triatomine abundance

Although environmental variables such as altitude, climate, vegetation type, and land use all affect triatomine presence at a small spatial scale [28], these likely do so indirectly, by increasing the availability of suitable habitats for triatomine hosts. From sylvatic habitats, triatomines can spread over long distances through passive dispersal, as eggs, nymphs and adults may be transported in human clothes, mammalian hair or bird feathers [12,29,30].

Our study identified key factors explaining *R. ecuadoriensis *abundance in sylvatic habitats such as the type of nest, nest height from ground and nest distance to the nearest house. These results agree with previous results performed in the coastal region of Ecuador (Manabí Province) [16]. Squirrel nests are commonly infested with triatomines, probably due to the size and loose construction of the nest, the size of the animal (as a food source), and the animals' constant occupancy of the nest. These nests were found at a height up to 20 meters, but were more commonly at about 8 meters. Such high height seems to be related to ecological preferences of squirrels which generally look for large, tall trees providing more nesting places, a better thermal insulation, and lower risk of predation [31]. The distribution of the squirrel species *S. stramineus *in Ecuador seems to coincide with that of *R. ecuadoriensis *(from 0 to 2000 m.a.s.l., central and southern coast and occidental Andes slope of Ecuador) [32]. The role of this squirrel species in the dispersal of sylvatic populations of *R. ecuadoriensis *needs to be further explored, as it may have important implications for the long-term control of Chagas disease in Ecuador. Mouse and rat nests were also significantly infested. They were more numerous than squirrel nests, but of smaller size. In southern Ecuador, *Rattus rattus *has been considered an important reservoir of *T. cruzi *[33] and implicated as a possible link of sylvatic *T. cruzi *populations in domestic transmission [34]. In addition, field observations indicate that squirrels are highly mobile, attracted by the crops, and frequently abandon nests, which in turn become occupied temporarily or permanently by rats. This behavior of nest re-users could explain the high *T. cruzi *infection rate found in rats and reinforces the possible role of these mammalian hosts as an important link between sylvatic and domestic transmission cycles. Finally, our results corroborate previous reports of the presence of *R. ecuadoriensis *in nests of the fasciated wren (Troglodytidae: *Campylorhynchus fasciatus*) whose large and loosely knitted nests provide adequate conditions for this triatomine species [16-18].

Within a given community, we found a significant correlation between triatomines abundance in sylvatic habitats and distance from human dwellings. Locations near homes probably offer more food sources, which may help triatomines to survive in deforested areas. As the distance from triatomine source sites contribute to bug establishment rate [35], there are important implications of knowing triatomine source sites for controlling house re-infestations after spraying. A study on re-infestation processes in Argentina suggests that Chagas disease control program should consider potential sources of triatomines up to 1,500 m around the dwelling to reduce adult invasion [36].

We also observed higher triatomine infestation rates in areas with water sources and vegetation that provides animal nesting places and food. Deforestation leads to a decrease in wild vertebrate populations, which could in turn decrease populations of sylvatic triatomines, but residual populations could feed on opportunistic marsupials and rodents. In addition, starving triatomine adults from large colonies would fly into nearby homes, leading to an increased risk of disease transmission [37].

### Infection rates with trypanosomes in *R. ecuadoriensis*

Our study showed high infection rate of *R. ecuadoriensis *with *T. cruzi *thereby confirming the role of this triatomine as a major vector of the Chagas disease in southern Ecuador [9,13]. In addition, the high infection rates observed with *T. rangeli *are also consistent with previous reports [24,25] and could constitute confounding factors in epidemiological studies.

Fifty six samples of *T. cruzi *from sylvatic triatomines collected during this study were genotyped and determined as belonging to the Discrete Typing Unit (DTU) *T. cruzi *I (TcI) [34]. Microsatellite analyses grouped most samples (n = 53) as part of a sylvatic genotype population that appeared genetically separated from a cluster formed by the domestic/peridomestic population. Although limited, genetic flow among sylvatic and domestic/peridomestic trypanosomes was evident. As mentioned before, the complex interactions of vectors and host, especially in sylvatic environments, could play an important role in transmission of the disease in the studied area. To elucidate this scenario, a longitudinal study that compares the original parasite isolates with those isolated from triatomines collected after control intervention needs to be conducted. Also the role of synanthrophic mammals, which could connect both environments, must be clarified.

### Interactions between sylvatic and non-sylvatic *R. ecuadoriensis *populations

Recent morphometric analyses of wings and antennal phenotype indicated that sylvatic and domicile/peridomicile populations of *R. ecuadoriensis *shared phenotypic characteristics in Loja province suggesting a continuous exchange of individuals between both environments [38]. Although triatomine re-infestation found after spraying campaigns in southern Ecuador (Grijalva et al. unpublished data) could be due to the presence of remnant populations of bugs that have not been killed by the insecticide, we hypothesize that the arrival of sylvatic populations to dwellings may be an important re-infestation source.

## Conclusions

Vectorial transmission of Chagas disease relies on the presence of triatomines in domestic habitats. Although conclusive evidence does not yet exists that the post-spraying house reinfestation is due to sylvatic bug migration, we propose that the widespread presence of sylvatic populations of *R. ecuadoriensis *could jeopardize long-term control of Chagas disease transmission in southern Ecuador. If this information is corroborated, alternative control measures should be implemented in areas with documented presence of sylvatic *R. ecuadoriensis *populations. Control interventions should include systematic and periodic active and passive entomological surveillance, insecticide spraying, house improvement programs, insecticidal paints [39], community education, and monitoring of sylvatic triatomine populations.

## List of Abbreviations

DTU: Discrete Typing Unit; GLMs: Generalized Linear Models; AIC: Akaike's Information Criterion; LRT: Likelihood ratio tests.

## Competing interests

The authors declare that they have no competing interests.

## Authors' contributions

MJG: conceived of the study, and participated in its design and coordination, performed data analyses and helped to draft the manuscript. VS-D: contributed to the sampling design, conducted sylvatic triatomine collection, data analysis and drafted the manuscript. AGV: directed entomological collection, triatomine identification, data analyses and helped to draft the manuscript. SO conducted parasitological analyses and helped to draft the manuscript. OD: Conceived the study, contributed to the design and data analyses and helped to draft manuscript. All authors read and approved the final manuscript.
